# Stalking influenza by vaccination with pre-fusion headless HA mini-stem

**DOI:** 10.1038/srep22666

**Published:** 2016-03-07

**Authors:** Sophie A. Valkenburg, V. Vamsee Aditya Mallajosyula, Olive T. W. Li, Alex W. H. Chin, George Carnell, Nigel Temperton, Raghavan Varadarajan, Leo L. M. Poon

**Affiliations:** 1 HKU-Pasteur Research Pole, School of Public Health, HKU Li Ka Shing Faculty of Medicine, The University of Hong Kong, Hong Kong; 2 Center of Influenza Research and School of Public Health, The University of Hong Kong, Hong Kong; 3 Molecular Biophysics Unit, Indian Institute of Science, Bangalore, India; 4 Viral Pseudotype Unit, School of Pharmacy, University of Kent, Kent, United Kingdom

## Abstract

Inaccuracies in prediction of circulating viral strain genotypes and the possibility of novel reassortants causing a pandemic outbreak necessitate the development of an anti-influenza vaccine with increased breadth of protection and potential for rapid production and deployment. The hemagglutinin (HA) stem is a promising target for universal influenza vaccine as stem-specific antibodies have the potential to be broadly cross-reactive towards different HA subtypes. Here, we report the design of a bacterially expressed polypeptide that mimics a H5 HA stem by protein minimization to focus the antibody response towards the HA stem. The HA mini-stem folds as a trimer mimicking the HA prefusion conformation. It is resistant to thermal/chemical stress, and it binds to conformation-specific, HA stem-directed broadly neutralizing antibodies with high affinity. Mice vaccinated with the group 1 HA mini-stems are protected from morbidity and mortality against lethal challenge by both group 1 (H5 and H1) and group 2 (H3) influenza viruses, the first report of cross-group protection. Passive transfer of immune serum demonstrates the protection is mediated by stem-specific antibodies. Furthermore, antibodies indudced by these HA stems have broad HA reactivity, yet they do not have antibody-dependent enhancement activity.

Vaccination is one the most effective means for public health control of infectious diseases. A vaccine against influenza virus has been available for over 70 years, yet influenza still causes epidemics or pandemic with substantial morbidity and mortality. The protective responses induced by current human influenza vaccines still primarily depend on vaccine-induced neutralizing antibodies (nAbs) against the HA head^1^. However, the continually evolving influenza virus evades herd immunity induced through natural infection and vaccination by means of antigenic drift and shift. These antigenic drift and shift events render vaccine stockpiling unviable in case of an outbreak or pandemic. In addition, a major shortcoming of current influenza vaccines is its long production time because of existing egg-based or cell-based vaccine manufactory pipelines. Thus, there is a need for novel influenza vaccines with increased breadth of protection and potential for rapid production and deployment.

The HA of influenza viruses, except those recently detected from bats, can be classified into 2 phylogenetic groups based on sequence conservation, group 1 (H1, H2, H5, H6, H8, H9, H11, H12, H13 and H16) and group 2 (H3, H4, H7, H10, H14 and H15)^2^. The HA protein on the virion surface is a trimer, in a pre-fusion state as HA0, and is then cleaved by cellular proteases to HA1 and HA2^3^. The majority of the HA1 chain forms the globular head, which contains the receptor-binding site (RBS). The accumulation of amino acid changes and glycosylation sites in the globular head domain might render pre-existing antibody responses obsolete. By contrast, the HA stem, predominantly comprised of the HA2 domain, is structurally conserved across HA subtypes^4^,^5^. The conserved stem domain contains a greater proportion of vulnerable sites targeted by broadly neutralizing antibodies (bnAbs) than the variable head domain^6^. Importantly, anti-stem broadly neutralizing antibodies (bnAbs) are detectable in some individuals at a low level, suggesting they can be induced naturally by infection and optimized by vaccination. Thus, structure-guided immunogen design targeting conserved, vulnerable sites in the stem of influenza HA might be a promising approach to ‘universal’ influenza vaccine development.

Humoral responses against the stem domain post infection or vaccination are weak relative to those against the head domain. Thus, eliciting cross-protective, stem-directed bnAbs remains challenging^7^. Different strategies, such as headless HA virus^8^, prime-boost with a chimeric HA protein^9^,^10^, sequential infection with different influenza subtypes^11^, or polypeptide mimics to the HA stem^12^,^13^,^14^,^15^,^16^, have been employed to elicit bnAbs antibodies towards this domain. Animals immuninized by these new strategies are shown to be protected from challenges with viruses that are of the same HA subtype and/or group (e.g. H1 against H5). However, *in vivo* data showing protections of a group 1 HA stem vaccine against a lethal challenge of a group 2 HA virus or vice versa, have not been demonstrated or were unsuccessful. In this study, using our newly developed H5-mini stem polypeptide and our previously reported H1-mini stem as immunogens^12^, we describe the use of a group 1 HA mini-stem to induce protection against both group 1 and group 2 viruses.

## Results and Discussion

Highly pathogenic H5N1 virus is endemic in some countries. We therefore developed and tested the protective efficacy of a novel H5-based HA stem immunogen designed from influenza A H5N1 (VN/04: A/Viet Nam/1203/04) (GenBank Accession: AAW80717.1) virus. We also characterized our previously designed H1 mini-stem (PR/34: A/PR/8/34)^12^ in a mouse model against heterologous influenza virus challenge, and evaluated the vaccine mediated antibody responses. The recombinant protein vaccine was administered in combination with AddaVax, a squalene-based oil-in-water nanoemulsion similar to MF59 and AS03 adjuvants, which are licensed for human use in Europe for influenza vaccines^17^.

**Figure 1:**
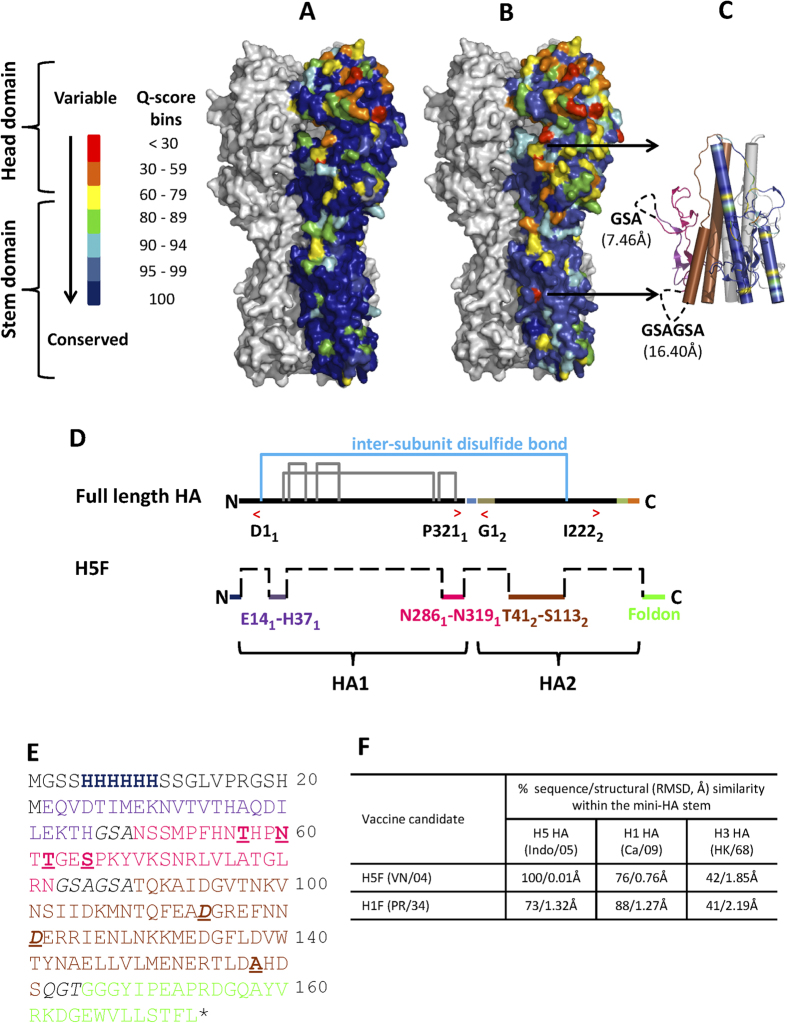
Design of HA mini-stem from HPAI H5N1 A/Viet Nam/1203/2004 HA. **(A)** All full-length H5N1 HA sequences isolated from human hosts (n = 215) were analyzed and the conservation score (Q-score) obtained from a multiple sequence alignment was mapped onto a monomer of the surface representation of the H5 HA A/Viet Nam/1203/2004 trimer (PDB ID: 2FK0), and the remaining HA in light gray. **(B)** Conservation analysis was extended to include all avian influenza H5 HA sequences (n = 2427) **(C)** The conserved HA stem fragments included in H5F (cartoon representation). One monomer was colored based on the Q-score. The second monomer was colored as described in (**E**), whilst the third monomer is shown in light gray. Dashed lines represent connecting linkers. Schematic of **(D)** full-length H5 HA and ‘headless’ HA stem-fragment, H5F. Intra-subunit (solid lines, black) and inter-subunit (solid line, blue) disulfide bonds are shown. The polybasic cleavage site (blue), fusion peptide (tan), trans-membrane domain (dark green) and the cytoplasmic tail (orange) are indicated. Red brackets (</>) are the start/stop sites of each subunit in the crystal structure (PDB ID: 2FK0). The crystal structure numbering was followed here. Dashed lines indicate linkers. A synthetic trimerization motif, foldon (light green), was appended at the C-terminus of the disulfide-free HA mini-stem to promote oligomerization. The expression vector derived N-terminal His-tag (dark blue) that facilitates a facile, one-step Ni-NTA affinity purification is also indicated. **(E)** Sequence of H5F colored according to the schematic in panel **(D)**. Site-specific mutations (bold and underlined) were introduced to prevent protein aggregation. Aspartate (**D**) mutations (bold, italics and underlined) were introduced to destabilize the low-pH conformation of HA. **(F)** The sequence conservation in the HA stem fragments between the vaccine candidates and influenza virus challenge strains was determined by pairwise sequence alignment (http://www.ebi.ac.uk/Tools/psa/emboss_stretcher/). The structural similarity (main chain) between HA stem fragments across different influenza virus strains was calculated by a topology independent comparison of modeled structures using the CLICK server (http://mspc.bii.a-star.edu.sg/minhn/output/143882270252.html). **(A–C)** were rendered using PyMOL.

Conserved residue patches in the HA stem comprising the ‘antigenic signatures’ of bnAbs were identified by a comprehensive H5 HA sequence (n = 2642) conservation analysis (Fig. 1A,B). An epitope focused stem immunogen, H5HA10-Foldon (hereafter referred to as H5F), was designed by analyzing the interaction network within H5 HA (VN/04)^18^ and mapped by generating an all-atom residue contact matrix using the in-house software PREDBURASA^19^. H5F consists of three stem derived fragments; HA1: 14–37, 286–319 and HA2: 41–113 (Fig. 1D,E). These fragments collectively comprise a significant fraction of the antibody (Ab) footprint of the bnAb CR6261, and minimally perturbed the pre-fusion HA stem interactions (Fig. 1C). However, protein minimization exposes a previously buried hydrophobic patch within the full-length HA, that can potentially lead to aggregation. Therefore, we re-surfaced this HA1 region with polar amino acid substitutions: I297T, I300T, and I302N. Also, the surface exposed, single cysteine residue (HA1 C305) in the HA mini-stem that can potentially form a non-native, inter-molecular disulfide bond was mutated to serine. We introduced aspartate mutations (HA2 V66D and L73D) in the HA mini-stem to destabilize the low-pH conformation of HA (Fig. 1E) as described previously^20^. The individual stem fragment termini were connected using soluble, flexible GSA linkers (Fig. 1E). A synthetic, trimerization motif “Foldon”^21^ was fused at the C-terminus of the HA mini-stem to promote oligomerization (Fig. 1D,E). Analysis of the sequence conservation and structural homology of the designed HA mini-stems with the stem domain of viruses used for challenge in this study (H5N1: A/Indo/05 (Indo/05), pdm H1N1/2009: A/California/04/09 (Ca/09) and mouse adapted H3N2: A/HK/68 (HK/68)), showed that mimicking the pre-fusion HA stem interactions to present a ‘functional’ binding surface is an essential pre-requisite to elicit broadly, cross-protective immune response(s), highlighting the need to present ‘conformational’ rather than ‘sequential’ epitopes to the immune system (Fig. 1F). The designed HA mini-stem was expressed in *E. coli* and recovered by a single, affinity-purification step. Biophysical characterization of H5F indicated a stable, well-folded, α-helical protein (Figure S1A–C), which formed a homogenous trimer in solution (Figure S1D). Proteins expressed in a prokaryotic system lack post-translational modifications (PTMs), therefore our *E. coli* expressed HA mini-stem lacks glycosylation and may have enhanced accessibility of conserved stem epitopes resulting in increased cross-reactive Ab responses relative to native protein immunization^22^.

Stem-directed bnAbs are robust probes for evaluating HA conformation^23^,^24^,^25^, since their epitopes are disrupted in the extended, low-pH conformation of HA. H5F bound the stem-directed, conformation selective bnAbs with high affinity (11.6 ± 1.4 to 43.5 ± 10.7 nM) (Table S1, Figure S2). The antigen-antibody complex of H5F with CR6261-IgG was specifically pulled down with Protein G beads (Figure S3). These results indicate that HA mini-stem closely mimics the native, pre-fusion conformation of HA. Additionally, the HA mini-stem was resistant to diverse, pharmaceutically relevant stress conditions and retained binding to CR6261-IgG (Table S2, Figure S1E). The physical and chemical stability of HA mini-stem suggests it can be transported and stored without requiring a cold-chain, facilitating stockpiling as well as deployment during an outbreak in low-resource settings.

**Figure 2:**
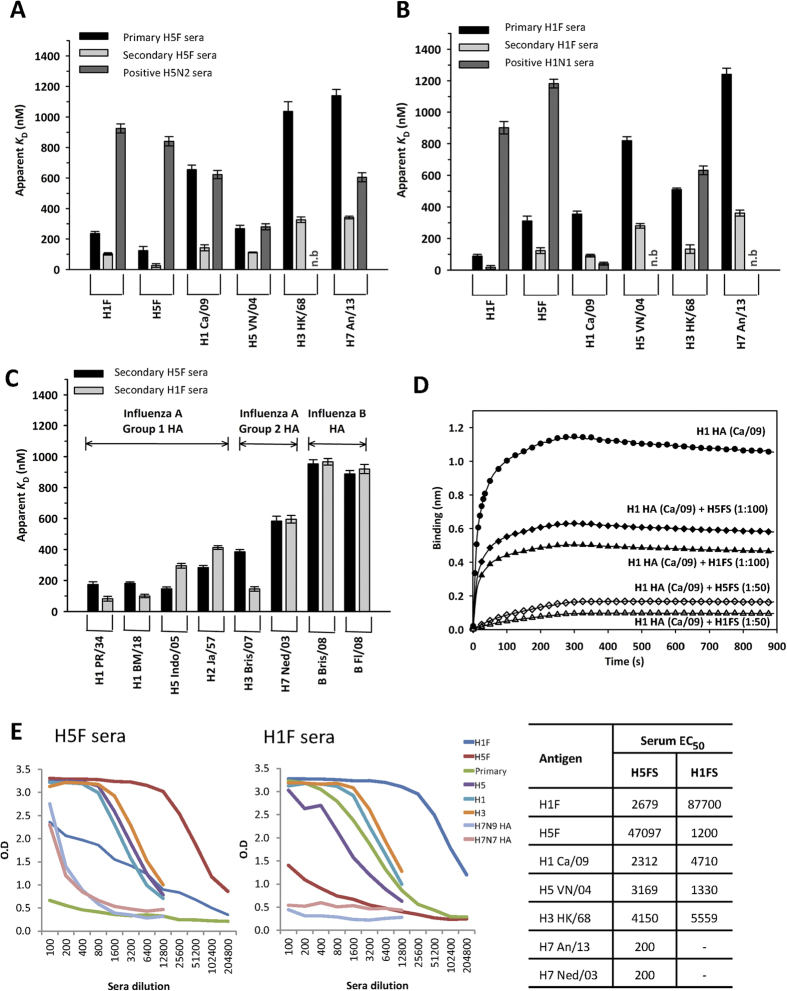
The HA mini-stem vaccines elicit broad, cross-reactive neutralizing Abs in mice. The binding of sera (total IgG) to HA proteins was probed by ELISA/BLI, from day 21 post primary (pooled n = 8) or secondary vaccine dose (pooled n = 20). **(A–C)** The sera Abs (total IgG) were captured using Protein G (ProG) biosensors and the kinetics of binding to HA proteins was determined using an Octet RED96 instrument. Lower equilibrium dissociation constant (*K*_D_) indicates higher affinity. Convalescent sera from mice that recovered from a sub-lethal H5N2 (Wigeon/07) or H1N1 (pdm Ca/09) virus challenge was used as positive controls. The BLI determined sera reactivity profile of primary and secondary **(A)** H5F and **(B)** H1F immune sera (n.b. indicates no detectable binding). **(C)** The BLI determined sera reactivity profile of secondary immune sera against a panel of HAs from influenza A groups 1 and 2, and influenza B using BLI. **(D)** Immune sera competition with the bnAb CR6261 for binding to H1 HA (pdm Ca/09). CR6261 was captured on amine reactive biosensors and the interaction with full-length H1 HA (pdm Ca/09) was monitored by BLI using the Octet RED96 instrument. The binding of H1-HA (pdm Ca/09) protein to CR6261 was also probed after incubation (30 mins) of the protein with different sera dilutions (as indicated). The decreased binding of H1 HA (pdm Ca/09) to the immobilized CR6261 in the presence of vaccine sera indicated the existence of competing antibodies. Pooled serum (n = 5–20 mice) was used, experiments were repeated two to three times and performed in duplicate. (**E**) Vaccine sera tested in direct ELISA specific for different recombinant HA proteins.

The immunogenicity of adjuvantated HA mini-stem designed from HPAI H5N1 (VN/04) was assessed by extensive characterization of the sera from vaccinated mice. We also probed the Ab response elicited by H1HA10-Foldon (referred to as H1F), a previously characterized “headless” HA stem-fragment immunogen designed from influenza A H1N1 (PR/34) virus^12^. The breadth of Ab response elicited by HA mini-stem vaccination was probed by enzyme-linked immunosorbent assay (ELISA) and biolayer interferometry (BLI) (Fig. 2 and Fig. S4, Table S3) against an extensive panel of recombinant HA proteins. Two-dose vaccination with HA mini-stem significantly enhanced the magnitude of cross-reactive Ab responses relative to a single-dose (Fig. 2A,B). Furthermore, lower off-rates (*k*_off_) of secondary sera versus primary sera binding to HA indicates affinity maturation (Tables S4 and S5). Positive control sera from recovered mice with H5N2 (A/Eurasian Wigeon/MP4610/2007) or H1N1 (pdm Ca/09) virus challenge showed weak HA-stem binding and significantly lower HA cross-reactivity, which is consistent with the immunodominance of the hypervariable HA1 head domain during natural infection (Fig. 2A,B). The Abs elicited by the HA mini-stem after two immunizations showed broad HA cross-reactivity and bound group 1 HAs (H1/H5/H2) with high affinity (Fig. 2C,D).

The binding of HA mini-stem to the pan-influenza bnAb, FI6v3 (Table S1, Figure S2), and the potential for developing a broadly reactive antibody response by our vaccine motivated us to test the sera against influenza A group 2 HAs (H3/H7). Encouragingly, the Abs elicited by the designed immunogens also bound H3 and H7 HAs with modest affinity, and low-level binding to influenza B HA (Fig. 2C-E). Antibodies elicited by the HA mini-stem compete with the bnAb CR6261 for binding to H1N1 (pdm Ca/09) HA (Fig. 2D), therefore they may also inhibit virus replication by targeting the stem region.

A yeast surface display (YSD) library expressing HA fragments of heterologous influenza A H1 HA (pdm Ca/09) and H3 HA (HK/68) were used to determine whether these mini-stem can induce antibodies that are specific to the HA2 stem region (Figure S5). The HA mini-stem vaccine serum showed higher-level H1-YSD library binding than H1N1 (pdm Ca/09) convalescent sera (Figure S5A). The binding of HA mini-stem vaccine serum for the H3-YSD library was also higher than H1N1 (pdm Ca/09) convalescent sera, which had no detectable binding (Figure S5D). Sequencing of HA-YSD clones bound by H1F and H5F serum indicated an immunodominant antibody response towards the HA stem (Figure S5H), therefore our vaccine successfully stimulates stem-specific antibodies.

**Figure 3:**
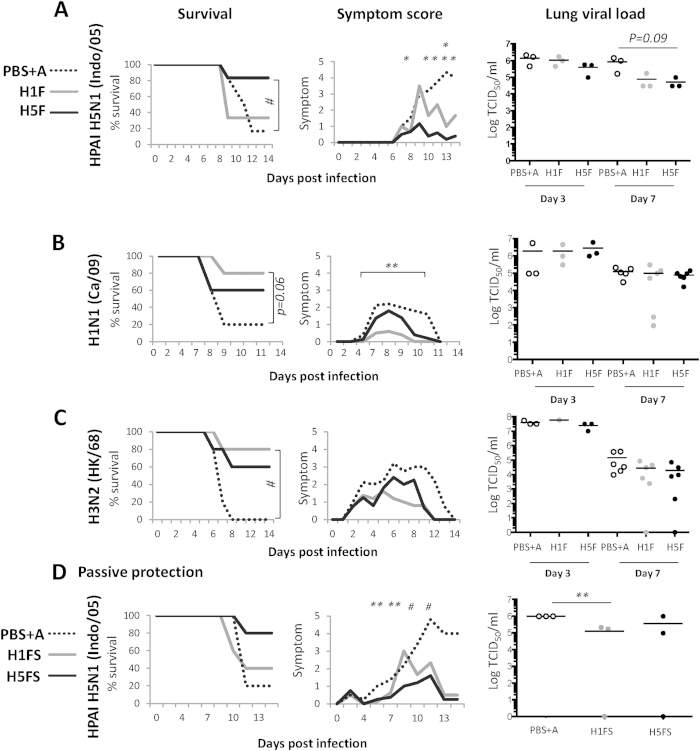
HA mini-stem immunization provides robust protection against lethal heterologous virus challenge in mice. Female BALB/c mice were vaccinated twice at 3 weeks interval by the intramuscular (i.m.) route with 20 μg of HA mini-stem adjuvanted with Addavax. PBS with Addavax (PBS + A) vaccinated mice were used as a negative control. Vaccinated mice were challenged 21 days after the secondary immunization with **(A)** HPAI H5N1 (Indo/05, 30TCID_50_), **(B)** H1N1 (pdm Ca/09, 1.36 × 10^6^ TCID_50_) or **(C)** H3N2 (HK/68, 2.5 × 10^6^ TCID_50_) virus. Survival and symptom severity were monitored for 14 days post virus challenge (n = 5). Lung viral loads at day 3 and day 7 were determined by standard TCID50 on MDCK cells (n = 3–6). **(D)** Passive transfer of immune sera (day 21 after the secondary immunization, heat inactivated, pooled n = 20 mice). 500 μl of serum was given intraperitoneally (i.p.) prior to HPAI H5N1 (Indo/05, 30TCID_50_) virus challenge of naïve BALB/c mice (n = 8). Survival, symptom severity (n = 5), and day 7 viral loads (n = 3) were determined. Statistics for survival was determined by a log-rank test. Statistics for symptom severity and lung viral loads versus PBS + A controls were determined Student’s t-test. (P-values: ^#^<0.05, ^*^<0.01, ^**^<0.001).

The HA mini-stem immunized mice were challenged with heterologous influenza viruses: HPAI H5N1 (Indo/05), pandemic H1N1 (pdm Ca/09) and H3N2 (HK/68) to determine the *in vivo* protective efficacy of stem-specific antibodies. H5F (VN/04, clade 1) conferred robust protection against an unmatched, heterologous HPAI H5N1 (Indo/05, clade 2.1) virus challenge with minimal weight loss (<5%) and no signs of clinical morbidity (especially no neurological symptoms) (Fig. 3A), whilst H1F vaccine (PR/34) conferred partial heterosubtypic protection against HPAI H5N1 (Indo/05) virus challenge. Further, the mice immunized with H1F and H5F survived the lethal pandemic H1N1 (pdm Ca/09) virus challenge with reduced symptoms and reduced symptom severity (Fig. 3B).

Previous attempts to elicit cross-group protection via HA stem-focused immune response have had limited success with even modest challenge doses, such as the H5-chimeric HA vaccine model for H3N2 challenge^10^. Encouragingly, immunization with our H1F and H5F HA mini-stem, designed from group 1 influenza A viruses, conferred significant protection against a lethal influenza A group 2 H3N2 (HK/68) virus challenge (Fig. 3C), with 80% survival of H1F vaccinated mice. Whilst protection was clearly evident from our vaccine by increased survival and reduced symptom severity from each challenge experiment, vaccination did not significantly alter viral loads at day 3 post-infection. Encouragingly, at day 7 post-infection, lower viral loads or complete clearance was observed in few of the vaccinated mice (not statistically significant). However, the lung inflammation profile was not significantly different in vaccinated mice as compared to the naive group (Figure S6), in terms of neutrophil and macrophage influx (Figure S6A,B), alternative activated macrophages (Figure S6C), protein leakage within the lung (Figure S6D) or overall histology by H&E staining (Figure S6E). Therefore, whilst our vaccine is clearly protective, the mode of protection from our mini-stem vaccine is not directly related to limiting virus replication or inflammation in the lung. This is consistent with recent studies wherein stem-directed Abs although protective from mortality, failed to prevent infection^16^ or reduce lung viral loads^15^.

Vaccine protection is antibody mediated, as passive transfer of vaccine serum prior to lethal HPAI H5N1 (Indo/05) virus challenge increased survival (for homologous H5F vaccination), and reduced symptom severity (Fig. 3D). The lung viral loads were reduced by 8-fold for H1F sera and 3-fold for H5F sera, with viral clearance in 1 out of 3 mice for both the H1F and H5F groups. This increased protective effect on viral loads in passive transfer relative to active vaccination, may be due to the volume of sera used for transfer (500 μl) or possible interference from other vaccine specific immune responses during vaccination itself.

**Figure 4:**
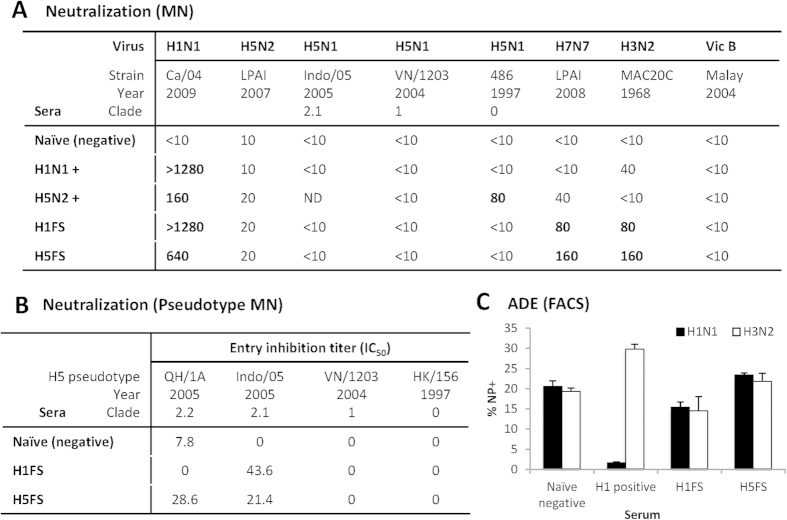
HA-stem specific neutralizing antibodies are CD4 dependent and do not augment viral entry. **(A)** The virus neutralization potency of ‘headless’ HA stem immunized mice sera was tested in a standard microneutralization assay against a panel of influenza viruses, and the lowest sera dilution able to neutralize 100TCID_50_ of a given virus is reported. Naïve mouse sera was used as a negative control, and convalescent sera from H1N1 (pdm Ca/09) or H5N2 (LPAI, Wigeon/07) virus challenge were used as positive controls. Experiments were performed at least 3 times. **(B)** *In vitro* neutralization activity of HA mini-stem vaccine serum against a panel of H5 pseduotyped virus particles. IC_50_ titer is the reciprocal of sera dilution at which half-maximal neutralization was observed. IC_50_ titer of zero indicates no detectable neutralization. **(C)** Antibody dependent enhancement was assessed by H1N1 (pdm Ca/09) or H3N2 (HK/68) infection of Raji cells in the presence of vaccine sera, convalescent sera or naïve mouse sera for 6 hours. Influenza NP expression of Raji cells was assessed by flow cytometry. % NP expression is shown, experiment was perfomred twice, in triplicate.

We next examined the neutralizing activity of HA mini-stem induced antibodies against a panel of viral subtypes to probe the mechanism of vaccine protection. In contrast to earlier studies^10^,^16^,^26^,^27^, neutralization of unmatched viral subtypes was observed in a stringent microneutralization (MN) assay (Fig. 4A), such as against H1N1 virus for heterologous H5F serum (1:640) and unmatched H1F serum (>1:1,280). The HA mini-stem antisera also showed neutralization activity (1:80–1:160) against influenza A group 2 (H3N2/H7N7) viruses from both H1F and H5F vaccine groups. The heterologous reactivity of the serum is consistent with a level of heterosubtypic protection offered by the vaccine. However, neutralization of influenza H5 viruses (group 1) from multiple clades could not be detected in the MN assay, including convalescent H5N2 (A/Eurasian Wigeon/MP4610/2007) serum for the homologous H5N2 virus. Other studies have also had difficulty in detecting H5-specific neutralisation^16^,^28^,^29^. In the more sensitive pseudotype based microneutralization assay (pMN), the HA mini-stem vaccine sera showed detectable activity against unmatched H5 viral strains, Indo/05 (clade 2.1) and QH/1A (clade 2.2) (Fig. 4B), whilst there was no activity for the vaccine homologous strain VN/1203 (clade 1).

The levels of neutralization observed in the MN and pMN assay agree with our *in vivo* mouse data that HA mini-stem immunized sera may not control initial virus replication, as high viral loads are reached at day 3 post infection (Fig. 3A–C). It is possible that a significant fraction of HA mini-stem induced antibodies are non-neutralizing. Interestingly, the lack of correlation between neutralizing antibody titer and protection was also recently observed in animals immunized by HA-stem nanoparticles^16^. The findings from this previous study and those from us suggest that HA-stem specific, non-neutralizing antibodies may also play a role in heterosubtypic protection to influenza virus. Non-neutralizing antibodies may be protective by recruiting other immune factors or cells for ADCC and complement^15^,^30^,^31^, altering virus fusion^32^, assembly or interfering with the virus life cycle^33^.

We also hypothesize that subtle differences between the neutralization epitopes of the HA stem among different viral subtypes may explain the disparate neutralization efficacy of the HA mini-stem polyclonal sera against the tested viruses. H5F and H1F vaccination induced a polyclonal Ab response which bind multiple epitopes within the HA-stem, resulting in H1N1, H3N2 and H7N7 virus neutralization, whilst these same epitopes may not be present in H5 viruses. Thus, protection from HPAI H5N1 infection by vaccine serum (Fig. 3D), may use an alternate immune mechanism which is yet to be determined. Further study on the modes of protection of these stem-specific antibodies will be needed.

The HA mini-stem elicits high affinity, stem-specific antibodies with protective potential. Further immune sera characterization revealed that vaccination with Addavax does not skew the balance of the natural Ab response, as the IgG1/IgG2a ratio (which reflects the Th1/Th2 balance) is similar to convalescent H3N2 (HK/68) sera (Figure S7A). The IgA and IgM levels in the sera are comparable to those in unvaccinated mice (Figure S7B,C). Vaccination also results in a broadly binding early antibody response from day 7 after H3N2 infection, which is otherwise undetectable in unvaccinated mice (Figure S7D), and the response is maintained to at least day 28-post challenge (Figure S7E). Furthermore, the magnitude and avidity of vaccine (H5F and H5) and cross-reactive (H3) Ab response remained relatively unchanged over time (Figure S7F,H) up to 120 days post vaccination.

There have been some concerns over antibody dependent enhancement (ADE) for stem-specific antibodies, resulting in increased influenza infection of vaccinated animals^32^. Whilst no increase in lung viral loads was observed in vaccinated mice (Fig. 3), to ensure our vaccine did not increase influenza virus entry, an *in vitro* infection in the presence of immune sera was performed (Fig. 4C). Raji cell infection rate in the presence of naïve mouse sera was 20%, whilst H1N1 convalescent sera had a 1.5% infection rate, an impressive 13-fold reduction. Whilst H1FS had a modest 15.4% H1N1 infection rate (1.3-fold reduction). H5FS had relatively comparable infection rate as naïve mouse serum, and therefore did not inhibit heterologous H1N1/H3N2 virus entry. Furthermore, H1N1-convalescent sera potentially increased H3N2 infection rate by 1.5-fold, which was not observed for our vaccine sera.

Our experiments showed that HA mini-stem vaccination generated high-titer, protective Ab responses. Further investigations are required to determine in more detail the protective mechanisms of HA stem-specific antibodies, which may act by alternate mechanisms such as interruption of virus life-cycle during entry, fusion, replication or by recruiting host immunity via antibody-dependent cellular cytotoxicity (ADCC) or complement activation.

We successfully engineered a H5-based HA mini-stem, using only a quarter of the full length HA, therefore focusing the immune response to the conserved epitope of known bnAbs in the stem domain of HA. The H5 HA mini-stem protein folds as a trimer, in a pre-fusion conformation of the HA stem. The physico-chemically tolerant HA mini-stem immunogens are resistant to thermal denaturation and pH-dependent conformational switching, thus no cold chain is required for the vaccination schedule. The recombinant protein vaccine is purified from *E. coli*, and can therefore be readily mass produced. These are essential properties for the vaccine production pipeline, pre-pandemic stockpiling and large scale production. FluBlok, a recombinant trivalent-HA protein influenza vaccine expressed in baculovirus is already licenced by the FDA for human use (http://www.fda.gov/NewsEvents/Newsroom/PressAnnouncements/ucm335891.htm). This sets a precendent for the use of recombinant protein based anti-influenza vaccines in humans.

The HA mini-stem vaccination elicited broad, cross-reactive, neutralizing Ab responses that breached the HA group-specific barrier and conferred robust protection against lethal heterologous influenza A virus challenge from both group 1 and 2 lineages. Recently there have been other studies targeting the conserved HAstem using chimeric HA^10^, mini-HA stem^15^ or multivalent display on nanoparticles^16^. However, ours is the first study to demonstrate the *in vivo* protective efficacy of a group 1 vaccine, against both group 1 (H5N1 and H1N1) and group 2 (H3N2) viruses with impressive survival rates. Our study provides a promising strategy for developing a HA stem-based ‘universal’ influenza vaccine.

## Materials and Methods

### Sequence conservation analysis

All non-identical, full-length influenza H5 HA sequences isolated from human (n = 215) and avian (n = 2427) hosts deposited in a public database (http://www.ncbi.nlm.nih.gov/genomes/FLU/FLU.html) were analyzed. The sequences were clustered at 99% homology to avoid over-representation using CD-HIT (Cluster Database at High Identity with Tolerance)^34^ to filter-out a total of 867 (n = 60/807 from human/avian hosts) unique, representative H5 HA sequences. Subsequently, these sequences were multiply aligned using CLUSTAL^35^. The measure of residue conservation at every position in the sequence is given by the quality score (Q-score) in the alignment file. These scores were binned and mapped onto the crystal structure of H5 HA A/Viet Nam/1203/2004 [Protein Data Bank (PDB) ID: 2FK0]^18^.

### Gene synthesis and protein purification

The *E. coli* codon-optimized gene sequence of H5F was synthesized (GenScript, USA) with a stop codon at the 3′ end. Subsequently, the gene was cloned in-frame with the vector (pET-28a (+) (Novagen)) derived N-terminal His-tag between the NdeI (5′ end) and HindIII (3′ end) restriction sites. The HA mini-stem proteins used in this study were purified using a similar protocol as described previously^12^. The purified protein was dialysed (against PBS containing 1 mM EDTA (pH 7.4)) and concentrated to ~5 mg/ml. The protein identity was confirmed by ESI-MS (electrospray ionization-mass spectroscopy).

### Viruses and recombinant full-length HA proteins

Viruses and full-length influenza A HAs (Sino Biological Inc., Beijing, China) are listed as indicated; H1N1 A/California/04/2009 (pdm Ca/09), H1N1 A/Puerto Rico/8/1934 (PR/34), H1N1 A/Brevig Mission/1/1918 (BM/18), H2N2 A/Japan/305/1957 (Ja/57), H5N1 A/Viet Nam/1194/2004 (VN/04), H5N1 A/Indonesia/5/2005 (Indo/05), H3N2 A/Hong Kong/1/1968 (HK/68), H3N2 A/Brisbane/10/2007 (Bris/07), and H7N7 A/Netherlands//219/03 (Ned/03). Influenza B HAs (Sino Biological Inc., Beijing, China); B/Brisbane/60/2008 (Bris/08) and B/Florida/4/2008 (Fl/08).

### Circular dichroism

A Jasco J-715 C spectropolarimeter flushed with nitrogen gas was used to record circular dichroism (CD) spectra for all protein samples. The spectra were acquired at 25 °C at a scan rate of 50 nm/min using a 1 mm path-length quartz cuvette. The response time was set to 4 s with a bandwidth of 2 nm. The represented data was averaged over five consecutive scans and corrected for buffer (PBS, pH 7.4) contributions. Mean residue ellipticity (MRE) was calculated as described previously^36^.

Thermal denaturation of H5F (in PBS, pH 7.4) was followed by monitoring the CD-signal at 208 nm between 15–90 °C with a 1 °C/min gradient. The reversibility of thermal unfolding was determined by repeating the scan after cooling the heated sample.

### Fluorescence spectroscopy

A Jasco FP-6300 spectrofluorometer was used to acquire the intrinsic fluorescence measurements for all the protein samples at 25 °C. The samples were excited at a wavelength of 280 nm, and fluorescence emission was monitored from 300–400 nm. The excitation and emission slit widths of the spectrofluorometer were set at 3 nm and 5 nm, respectively. The represented data was averaged over five consecutive scans and corrected for buffer signals. Fluorescence spectra of the native protein were recorded in PBS (pH 7.4). The protein was denatured in 7 M guanidine hydrochloride (GdmCl) to record the unfolded state fluorescence.

### Size exclusion chromatography – Multi angle light scattering (SEC-MALS)

The oligomeric state of H5F under native conditions was determined at 25 °C by gel filtration chromatography on a Superdex-200 analytical gel filtration column (GE HealthCare) equilibrated with PBS (pH 7.4) at a flow rate of 0.5 ml/min. The column was calibrated using a broad range of molecular weight markers (GE HealthCare). Alternatively, for molecular weight determination, the Superdex-200 analytical gel filtration column (GE HealthCare) was connected with in-line UV, refractive index and triple-angle MALS scattering (miniDAWN TREOS, Wyatt Technology) detectors. ASTRA software was used for data analysis.

### Surface plasmon resonance (SPR)

Binding affinity of HA mini-stem (H5F) and full-length H5 HA (VN/04) (Sino Biological Inc.) to the stem-directed bnAbs; CR6261-IgG, F10-scFv and FI6v3-scFv was determined by SPR experiments using a Biacore 3000 optical biosensor (Biacore, Uppsala, Sweden) as described previously^12^, except the sensor surface was regenerated with multiple injections of 2 M MgCl_2_ after every binding event. The trimeric concentration of H5F and H5 HA (VN/04) were used to fit the data. The kinetic parameters were obtained by globally fitting the data to a 1:1 Langmuir interaction model using BIA EVALUATION software (version 3.1).

### Biolayer interferometry (BLI)

The binding affinity of H5F, after subjecting to diverse, pharmaceutically relevant stress condition(s) (as indicated in Table S2), to the conformation-specific bnAb CR6261 was determined by BLI using an Octet RED96 instrument (Pall ForteBio, CA, USA). CR6261 in PBST (PBS with 0.05% Tween 20, pH 7.4) was captured on amine reactive (AR2G) biosensor tips.

The competition of HA mini-stem immunized mice sera for binding to H1 HA (pdm Ca/09) was also determined using CR6261 immobilized on AR2G tips. The binding of H1 HA (pdm Ca/09) to CR6261 was probed in the absence and presence of different dilutions (as indicated) of HA mini-stem vaccinated mice sera.

The kinetic parameters for the binding of sera to HA proteins was determined by BLI. The total IgG was captured from the pooled mice sera using Protein G (ProG) biosensor tips. The IgGs from all test sera were captured to an equal thickness (~2.0 nm). A concentration series of HA proteins (2.0 μM, 1.0 μM and 0.5 μM) was used to determine the apparent equilibrium dissociation constant (*K*_D_). An unliganded sensor (reference sensor) was used during every interaction analysis as a control for non-specific analyte binding. The sensor surface(s) were regenerated after each binding experiment with 2 M MgCl_2_. The maximal binding capacity of the ProG tips was comparable after every regeneration event as tested by binding of a control sera (convalescent H1N1 (pdm Ca/09) sera) with 1.0 μM of H1 HA (pdm Ca/09).

The trimeric concentration of HA proteins were used to fit all the BLI data. All the traces were corrected for non-specific signals (if any) and processed using the ForteBio Data Analysis Software (v8.0) and fit globally using a simple 1:1 Langmuir interaction model.

### Pull-down assay

The specificity of H5F and CR6261 IgG complex was probed by a targeted IgG pull-down assay using Protein-G beads (GE HealthCare). H5F was incubated with CR6261 at different molar ratios for 2 h at 4 °C adjusted with PBS (pH 7.4) to a final volume of 30 μl. This was followed by the addition of Protein-G beads (10 μl) to capture IgG. The tubes were spun down at 3000 × g for 15 mins at 4 °C and the unbound supernatant was separated from the beads. After two rounds of washes (with PBS, pH 7.4), the antibody bound to the beads was eluted (10 μl of 100 mM Glycine.HCl, pH 3.0) and neutralized (2.5 μl of with 1 M Tris.HCl, pH 9.0). The unbound and eluted fractions were analyzed simultaneously by SDS-PAGE and stained with Coomassie.

### Mice

Female 6 week-old BALB/c mice were vaccinated twice 21 days apart via the intramuscular route with 20 μg of antigen in 100 μl of PBS, with 1:1 Addavax adjuvant (Invivogen), and challenged with influenza virus 21 days later. PBS with Addavax (PBS + A) vaccinated mice were used as a negative control. For influenza challenge, anesthetized mice were infected intranasally with 25μl of H3N2 HK/68 (A/HK/1/68-MA20C, 2.5 × 10^6^ TCID_50_), pandemic H1N1 Ca/09 (A/California/04/2009, 1.36 × 10^6^ TCID_50_) or HPAI H5N1 Indo/05 (A/Indonesia/05/2005, 30TCID_50_). Serum was collected by cardiac puncture at time-points as indicated. Lungs were collected for determining infectious viral lung titre by standard TCID_50_ assay on MDCK cells, and histology. For immune cell profiling, bronchoalveolar lavage (BAL, a washing of the lung) and mediastinal lymph nodes (MLN, which drain the lower respiratory tract) were collected at day 3 and day 7. All experiments that involved HPAI H5N1 were conducted in BSL3 laboratory. All experimental procedures were conducted in accordance with the standards of humane animal care by the criteria outlined in the “Guide for the Care and Use of Laboratory Animals” prepared by the National Research Council, USA (2011) and approved by the Committee on the Use of Live Animals in Teaching and Research, The University of Hong Kong (http://www.lau.hku.hk/content/ethics/ethics.htm).

### Microneutralization assay (MN)

Pooled serum (n = 5 controls, n = 20 vaccine groups) was heat inactivated for 90 mins at 56 ^o^C. Two-fold serial dilutions from 1:5 to 1:3200 of serum were prepared in virus medium (MEM, 100 U/ml penicillin and 100 μg/ml streptomycin). An equal volume of 200TCID_50_/35 μl of influenza virus was added to the sample dilutions (final serum dilution 1:10 to 1:1280) and incubated for 2 hours at 37 °C, 5% CO_2_. Then 35 μl of antibody/virus was added to MDCK cells and incubated for 72 hours at 37 °C, 5% CO_2_. Standard haemagglutination assay and visualization of cytopathic effect was used to measure virus inhibition.

### Pseudotype based microneutralization (pMN) assay

The HA mini-stem vaccine sera were tested in a pseudotype virus particle entry inhibition assay as described previously^37^,^38^. Serial dilutions of the test sera were incubated with 1 × 10^6^ relative luminescence units (RLUs) of pseudotypes/well in 96-well flat-bottomed white plates (Nunc) in a final volume of 50 μl at 37 °C for 1 h. After the incubation, 1.5 × 10^4^ HEK293T cells were added to each well. The plates were subsequently incubated for another 48 h at 37 °C. The luminescence signals were assayed using the Bright-Glo assay system (Promega). The half-maximal inhibitory concentrations (IC_50_) of entry inhibition were determined using SigmaPlot.

### Enzyme linked immunosorbent assay (ELISA)

Briefly, recombinant HA protein (Sino Biological Inc.) and HA-Foldon vaccine (80 ng/ml) were coated on ELISA plates (Nunc-Immuno MaxiSorp, NUNC) overnight. Following FBS blocking, and washing, diluted serum samples were bound for 2 hours, washed and detected by anti-mouse IgG1-HRP (Invitrogen) as the secondary antibody. TMB/peroxide was used as substrate and the reaction was stopped by addition of sulfuric acid (R&D systems), and absorbance read at 450 nm.

### Influenza A Haemagglutinin yeast surface display epitope mapping (HA-YSD)

H3 HA-YSD and H1 HA-YSD library were constructed as previously described^39^. Yeast surface expressing peptides were constructed from random digestion of the HA fragments derived from the H3N2 (HK/68) HA gene, and H1N1 (pdm Ca/09). Thirty individual clones that could reach each pooled sample in each library were randomly selected for sequencing study. Sequences were then aligned to the orginal H3 (HK/68) or H1 (pdm Ca/09) HA sequence.

### Immune cell profiling

Cells were subject to FcR blocking (anti-CD16/CD32, BD Bioscience), and then stained with one of three cocktails containing a panel of monoclonal antibodies for innate and adaptive immune cells (all Biolegend unless otherwise indicated), for 30 mins on ice. *Cocktail 1:* F4/80-PE, I-AE-PerCPCy5.5, CD11b-APCy7, Gr1-PECy7, IA8-APC, CD11c-FITC. *Cocktail 2:* CD3-APC, CD4-APCCy7, CD8-PerCPCy5.5, NK1.1/Dx5-FITC, γδT-PE and B220-PECy7. *Cocktail 3:* F4/80-PECy7, CD11b-APCCy7, I-AE-PerCPCy5.5, MMR-APC, IL-4 Rα-PE, iNOS-FITC (BD Bioscience).

For intracellular stains, cells were fixed in fixation/permeabilsation buffer (eBioscience) for 30 mins on ice, and stained with iNOS-FITC in Perm wash buffer (BD Bioscience). Cells were finally fixed with 100 μl of 4% PFA for 20 mins on ice, and then washed and stained with DAPI in PBS for 10 mins on ice. Samples were acquired by flow cytometry on a FACS LSR Fortessa and analyzed with FlowJo software.

### *In vitro* antibody dependent enhancement for immune sera

Raji cells (Promega) (1 × 10^5^) were incubated with 25 μl of immune H1F or H5F serum (from 21 days post secondary vaccination, heat inactvated, pooled n = 5), H1N1 convalescent sera or naïve mouse sera. Cells were infected with an MOI of 4 of H3N2 (HK68) or H1N1 (pdm Ca/09) virus in the presence of sera for 16 hours in MEM (1%P/S). Cells were then fixed (BD cytofix/cytoperm buffer), and stained with NP-FITC (Abcam) in BD permiabilisation buffer for 30 mins on ice. Samples were acquired by flow cytometry on a FACS LSR Fortessa and analyzed with FlowJo software.

## Additional Information

**How to cite this article**: Valkenburg, S. A. *et al.* Stalking influenza by vaccination with pre-fusion headless HA mini-stem. *Sci. Rep.* **6**, 22666; doi: 10.1038/srep22666 (2016).

## Supplementary Material

Supplementary Information
