# Supplementary material for: Stalking influenza by vaccination with pre-fusion headless HA mini-stem
Source: Sci Rep. 2016 Mar 7;6:22666. doi: 10.1038/srep22666 (PMC4780079; doi:10.1038/srep22666)
Supplement: Supplementary Information [file srep22666-s1.pdf]

Stalking influenza by vaccination with pre-fusion headless HA mini-stem

Sophie A Valkenburg[a,b], V Vamsee Aditya Mallajosyula[c], Olive TW Li[b], Alex WH Chin[b], George Carnell[d], Nigel Temperton[d], Raghavan Varadarajan[c]*, Leo LM Poon[b*]

[a] HKU-Pasteur Research Pole, School of Public Health, HKU Li Ka Shing Faculty of Medicine, The University of Hong Kong, Hong Kong

[b] Center of Influenza Research and School of Public Health, The University of Hong Kong, Hong Kong

[c] Molecular Biophysics Unit, Indian Institute of Science, Bangalore, India

[d] Viral Pseudotype Unit, School of Pharmacy, University of Kent, Kent, United Kingdom.

* Correspondence:

Raghavan Varadarajan

Molecular Biophysics Unit,

Indian Institute of Science (IISc),

Bangalore

India

Email: varadar@mbu.iisc.ernet.in

Leo LM Poon

School of Public Health,

The University of Hong Kong

Hong Kong

Email: llmpoon@hku.hk#

Table S1: H5F binds stem-directed bnAbs with similar affinity as full length HA. Kinetic parameters (mean±SD) for the binding of H5F and H5 HA (VN/04) to conformation specific bnAbs were determined by surface plasmon resonance (SPR).

**Immunogen**	**Ligand**[a]	k_{on} **($M^{-1}s^{-1}$)**	k_{off} **(s^{-1})**	K_D **(nM)**
	CR6261-IgG	$3.4±1.1×10^4$	$1.4±0.1×10^{-3}$	43.5±10.7
H5F	F10-scFv	$1.8±0.8×10^5$	$1.8±0.5×10^{-3}$	11.6±1.4
	FI6v3-scFv	$2.4±1.2×10^5$	$2.9±0.3×10^{-3}$	12.1±3.8
	CR6261-IgG	$1.4±0.2×10^5$	$9.1±0.1×10^{-4}$	6.7±0.8
H5 HA (VN/04)	F10-scFv	$3.7±0.2×10^5$	$1.3±0.3×10^{-3}$	3.1±0.5
	FI6v3-scFv	$2.9±0.5×10^5$	$2.4±0.8×10^{-3}$	8.4±1.2

[a] 750RU of each ligand was immobilized on an independent activated surface channel of a Biacore CM5 sensor chip.

Table S2: H5F retained binding to the stem-directed bnAb CR6261 after diverse stress condition(s) treatment. Kinetic parameters (mean±SD) for the binding of H5F to CR6261 were determined by biolayer interferometry (BLI).

**Stress condition**		**K_D (nM), CR6261[a] binding**			
**Temperature (°C)[b]**		**25**	**40**	**60**	**80**
		37.1±3.3	40.2±2.5	54.4±6.8	85.4±2.2
**pH[c]**		**8.0**	**7.4**	**5.0**	**4.0[d]**
		36.9±5.1	37.1±3.3	46.8±2.4	62.7±1.8
**Temporal stability at different storage temperature**	**T=days**	**3**	**7**	**14**	**21**
	**4°C**	39.4±4.1	38.3±1.6	39.8±6.4	42.3±4.4
	**25°C**	36.1±3.1	40.8±0.8	43.1±1.7	51.2±2.7
	**37°C**	40.8±0.9	41.2±1.9	54.3±6.9	133.5±8.2
**Freeze-thaw cycles**		**1**	**3**	**5**	**10**
		38.1±2.4	37.4±4.4	42.1±5.8	56.9±2.7

[a] CR6261 (ligand) was captured on amine reactive biosensor tips.
[b] H5F (analyte) was incubated at the indicated temperatures for 1h. The samples were then cooled and K_D was determined at 25°C.
[c] H5F was incubated in buffered solutions of indicated pH for 30-45 mins and binding to CR6261 was subsequently evaluated. The sample pH was measured before the binding experiments.
[d] H5 HA (VN/04) showed no detectable binding after incubation at pH 4.0.

Table S3: Antibodies elicited by the engineered HA stem-fragment immunogens showed broad cross-reactivity. The equilibrium dissociation constant (K_D) for the binding of sera (total IgG) to HA proteins was determined by BLI using the Octet RED96 instrument. The sera from mice immunized with the HA stem-fragment immunogens bound heterologous HAs with high-affinity (K_D values ≤ 250nM are highlighted in red). The convalescent sera exhibited weak cross-reactivity.

**Analyte**	**Apparent K_D (nM)**[a]					
	Primary H5F sera	Secondary H5F sera	H5N2+ sera[b]	Primary H1F sera	Secondary H1F sera	H1N1+ sera[c]
**Group 1 HAs (mini-stem)**						
H1F	236.4±13.6	102.1±7.9	925.4±29.6	88.7±11.3	17.7±11.4	900.7±39.3
H5F	124.2±26.8	26.1±11.9	840.7±30.3	311.6±30.4	123.4±18.6	1182.3±27.7
**Group 1 HAs (full-length)**						
H1 A/Puerto Rico/8/1934 HA	-[d]	174.2±17.8	-	-	82.3±15.7	-
H1 A/California/4/2009 HA	655.2±29.8	142.4±19.6	623.1±26.9	354.8±19.2	91.7±8.3	40.8±9.2
H1 A/Brevig Mission/1/1918 HA	-	181.7±9.3	-	-	100.2±10.8	-
H5 A/Viet Nam/1194/2004 HA	268.4±21.6	112.1±2.9	280.1±19.9	818.5±25.5	280.6±14.4	n.b[e]
H5 A/Indonesia/5/2005 HA	-	146.9±12.1	-	-	295.1±14.9	-
H2 A/Japan/305/1957 HA	-	283.4±13.6	-	-	412.9±12.1	-
**Group 2 HAs (full-length)**						
H3 A/Hong Kong/1/1968 HA	1036.4±63.6	326.4±18.6	n.b	510.1±9.9	133.1±26.9	632.2±27.8
H3 A/Brisbane/10/2007 HA	-	385.6±14.4	-	-	145.8±14.2	-
H7 A/Netherlands/219/2003 HA	-	583.6±31.4	-	-	595.4±24.6	-
H7 A/Anhui/1/2013 HA	1139.5±40.5	342.1±9.9	605.2±29.8	1241.1±38.9	361.3±18.7	n.b
**Influenza B HAs (full-length)**						
B/Brisbane/60/2008 HA (Victoria lineage)	-	953.2±26.8	-	-	966.7±21.3	-
B/Florida/4/2008 HA (Yamagata lineage)	-	888.3±21.7	-	-	920.1±29.9	-

[a] The K_D values are the Mean ± SD of triplicate experiments.

[b] anti-H5N2+ sera : pooled sera of mice recovered from H5N2 (Wigeon/07) infection.

[c] anti-H1N1+ sera : pooled sera of mice recovered from pandemic H1N1 (Ca/04) infection.

[d] - : not determined because of sera limitation.

[e] n.b : no detectable binding.

Table S4: The kinetic parameters obtained for the binding of H5F sera (primary and secondary) and H5N2 convalescent sera against HA proteins determined by BLI using the Octet RED96 instrument. The convalescent serum (total IgG) binds HA stem-fragment proteins with low affinity and exhibits limited cross-reactivity.

Analyte	Serum	k_{on} ($M^{-1}s^{-1}$)	k_{off} (s^{-1})	K_D (nM)[a]
**Group 1 HAs (mini-stem)**				
H1F	Primary	4.15×10^3	9.81×10^{-4}	236.4±13.6
	Secondary	6.88×10^3	7.01×10^{-4}	102.1±7.9
	H5N2+	0.71×10^3	6.51×10^{-4}	925.4±29.6
H5F	Primary	8.21×10^3	10.21×10^{-4}	124.2±26.8
	Secondary	1.78×10^4	4.64×10^{-4}	26.1±11.9
	H5N2+	0.37×10^3	3.11×10^{-4}	840.7±30.3
**Group 1 HAs (full-length)**				
H1 A/Puerto Rico/8/1934 HA	Secondary	3.45×10^3	6.01×10^{-4}	174.2±17.8
H1 A/California/4/2009 HA	Primary	1.25×10^3	8.19×10^{-4}	655.2±29.8
	Secondary	2.35×10^3	3.34×10^{-4}	142.4±19.6
	H5N2+	0.65×10^3	4.07×10^{-4}	623.1±26.9
H1 A/Brevig Mission/1/1918 HA		3.34×10^3	6.07×10^{-4}	181.7±9.3
H5 A/Viet Nam/1194/2004 HA	Primary	3.23×10^3	8.67×10^{-4}	268.4±21.6
	Secondary	4.15×10^3	4.65×10^{-4}	112.1±2.9
	H5N2+	1.61×10^3	4.51×10^{-4}	280.1±19.9
H5 A/Indonesia/5/2005 HA	Secondary	3.15×10^3	4.63×10^{-4}	146.9±12.1
H2 A/Japan/305/1957 HA	Secondary	1.24×10^3	3.51×10^{-4}	283.4±13.6
**Group 2 HAs (full-length)**				
H3 A/Hong Kong/1/1968 HA	Primary	0.88×10^3	9.12×10^{-4}	1036.4±63.6
	Secondary	1.26×10^3	4.12×10^{-4}	326.4±18.6
	H5N2+	n.b[b]	n.b	n.b
H3 A/Brisbane/10/2007 HA		1.25×10^3	4.82×10^{-4}	385.6±14.4
H7 A/Netherlands/219/2003 HA		1.13×10^3	5.16×10^{-4}	583.6±31.4
H7 A/Anhui/1/2013 HA	Primary	0.81×10^3	9.23×10^{-4}	1139.5±40.5
	Secondary	1.43×10^3	4.92×10^{-4}	342.1±9.9
	H5N2+	1.05×10^3	6.37×10^{-4}	605.2±29.8
**Influenza B HAs (full-length)**				
B/Brisbane/60/2008 HA (Victoria lineage)	Secondary	1.07×10^3	10.2×10^{-4}	953.2±26.8
B/Florida/4/2008 HA (Yamagata lineage)	Secondary	0.94×10^3	8.35×10^{-4}	888.3±21.7

[a] The equilibrium dissociation constant (apparent K_D) values are the Mean ± SD of triplicate experiments.

[b] n.b : no detectable binding.

Table S5: The kinetic parameters obtained for the binding of H1F sera (primary and secondary) and anti-H1N1 convalescent sera (highlighted in green) against HA proteins determined by BLI using the Octet RED96 instrument. H1F elicited cross-reactive, anti-HA stem antibodies. In contrast, the convalescent serum (total IgG) displayed limited cross-reactivity.

Analyte	Serum	k_{on} ($M^{-1}s^{-1}$)	k_{off} (s^{-1})	K_D (nM)[a]
**Group 1 HAs (mini-stem)**				
H1F	Primary	1.12×10^4	9.93×10^{-4}	88.7±11.3
	Secondary	3.01×10^4	5.32×10^{-4}	17.7±11.4
	H5N2+	0.37×10^3	3.34×10^{-4}	900.7±39.3
H5F	Primary	3.51×10^3	10.91×10^{-4}	311.6±30.4
	Secondary	5.79×10^3	7.14×10^{-4}	123.4±18.6
	H5N2+	0.47×10^3	5.56×10^{-4}	1182.3±27.7
**Group 1 HAs (full-length)**				
H1 A/Puerto Rico/8/1934 HA	Secondary	8.25×10^3	6.79×10^{-4}	82.3±15.7
H1 A/California/4/2009 HA	Primary	2.81×10^3	9.95×10^{-4}	354.8±19.2
	Secondary	7.76×10^3	7.11×10^{-4}	91.7±8.3
	H5N2+	6.88×10^3	2.81×10^{-4}	40.8±9.2
H1 A/Brevig Mission/1/1918 HA	Secondary	7.21×10^3	7.23×10^{-4}	100.2±10.8
H5 A/Viet Nam/1194/2004 HA	Primary	1.12×10^3	9.16×10^{-4}	818.5±25.5
	Secondary	1.46×10^3	4.11×10^{-4}	280.6±14.4
	H5N2+	n.b[b]	n.b	n.b
H5 A/Indonesia/5/2005 HA	Secondary	1.37×10^3	4.04×10^{-4}	295.1±14.9
H2 A/Japan/305/1957 HA	Secondary	1.74×10^3	4.04×10^{-4}	412.9±12.1
**Group 2 HAs (full-length)**				
H3 A/Hong Kong/1/1968 HA	Primary	1.67×10^3	8.52×10^{-4}	510.1±9.9
	Secondary	3.48×10^3	4.63×10^{-4}	133.1±26.9
	H5N2+	1.47×10^3	9.29×10^{-4}	632.2±27.8
H3 A/Brisbane/10/2007 HA	Secondary	3.41×10^3	4.97×10^{-4}	145.8±14.2
H7 A/Netherlands/219/2003 HA	Secondary	1.03×10^3	6.15×10^{-4}	595.4±24.6
H7 A/Anhui/1/2013 HA	Primary	0.91×10^3	11.31×10^{-4}	1241.1±38.9
	Secondary	1.17×10^3	4.23×10^{-4}	361.3±18.7
	H5N2+	n.b	n.b	n.b
**Influenza B HAs (full-length)**				
B/Brisbane/60/2008 HA (Victoria lineage)	Secondary	1.18×10^3	10.8×10^{-4}	966.7±21.3
B/Florida/4/2008 HA (Yamagata lineage)	Secondary	0.99×10^3	9.15×10^{-4}	920.1±29.9

[a] The equilibrium dissociation constant (apparent K_D) values are the Mean ± SD of triplicate experiments.

[b] n.b : no detectable binding.

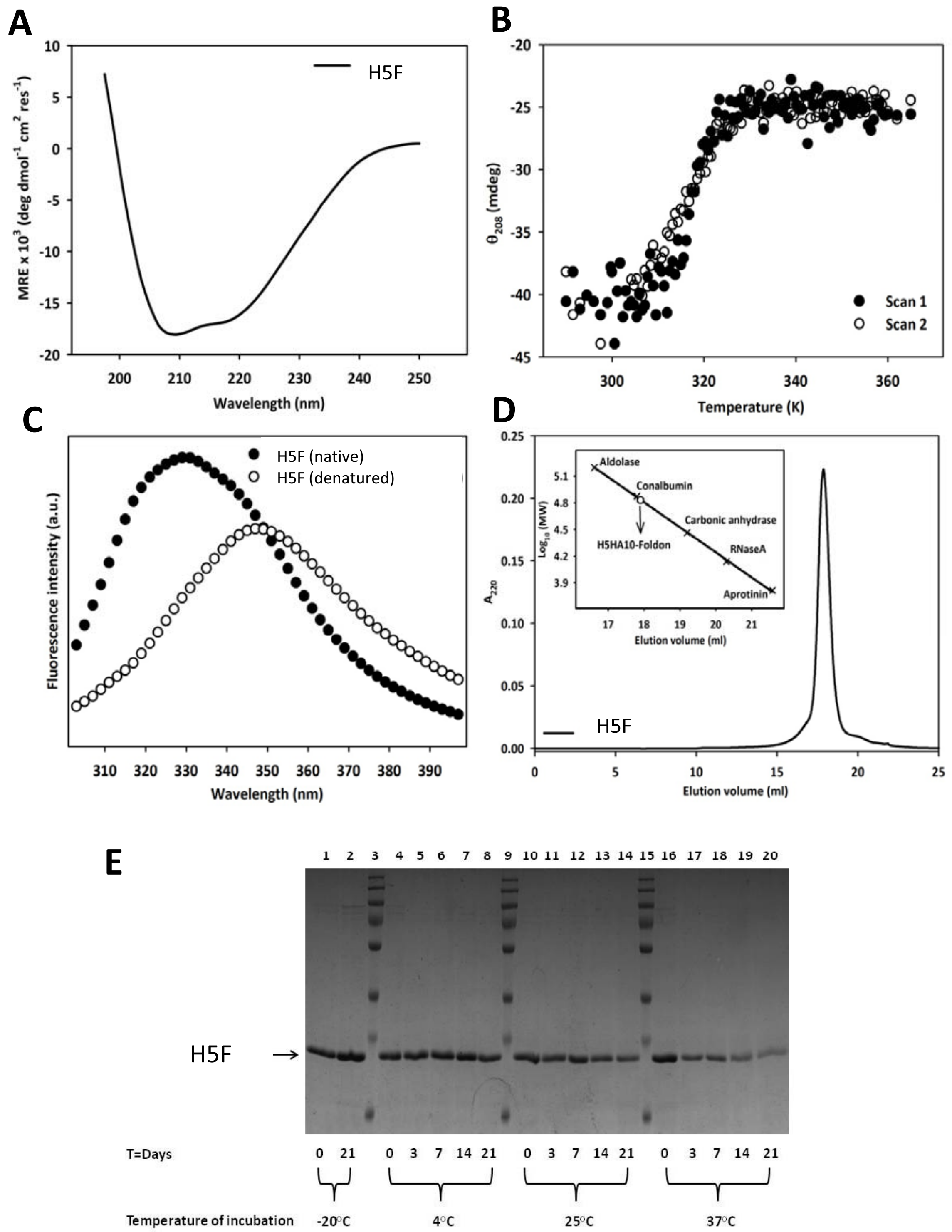

Figure S1: Biophysical and biochemical characterization of H5F. **(A)** The far-UV CD spectra of H5F indicated that the protein was well folded and α-helical, consistent with an extensive presence of helical HA2 subunit segments (external A-helix and the centrally located coiled-coil). **(B)** The thermal denaturation of H5F (~10μM) was monitored by CD at 208nm. The thermal unfolding of H5F was co-operative and reversible with an apparent thermal transition mid-point (T_m) of 318K (45°C). The consecutive unfolding traces of H5F overlapped well with each other indicating that protein unfolding was reversible. **(C)** The red-shift of the fluorescence emission maximum of H5F upon denaturation with GdmCl indicated the burial of aromatic, hydrophobic residues in the native, folded state. **(D)** The oligomeric state of H5F in solution was probed by analytical gel-filtration chromatography. The protein eluted as a homogeneous trimer. The molecular weight of H5F (◯) was calculated (67.6kDa) from the calibration curve (inset) of the S-200 column obtained using a broad range of markers (x). The theoretical molecular weight of H5F is 64.2kDa (21.4kDa × 3). The oligomeric state of the protein was also confirmed by SEC-MALS. The calculated molecular weight (6.523×10^4 (±8.97%) Da) was in good agreement with the theoretical molecular weight of a trimer. **(E)** Purified H5F was stored at different temperatures (as indicated). 10μl of protein was aliquoted at different time points (as indicated), flash frozen and stored at -70°C until further processing. All the samples were analyzed simultaneously on SDS-PAGE under non-reducing conditions and stained with Coomassie. H5F was stable for up to 3 weeks at 4°C and 25°C. Even at a higher storage temperature (37°C), only slight degradation of the protein is observed after 3 weeks.

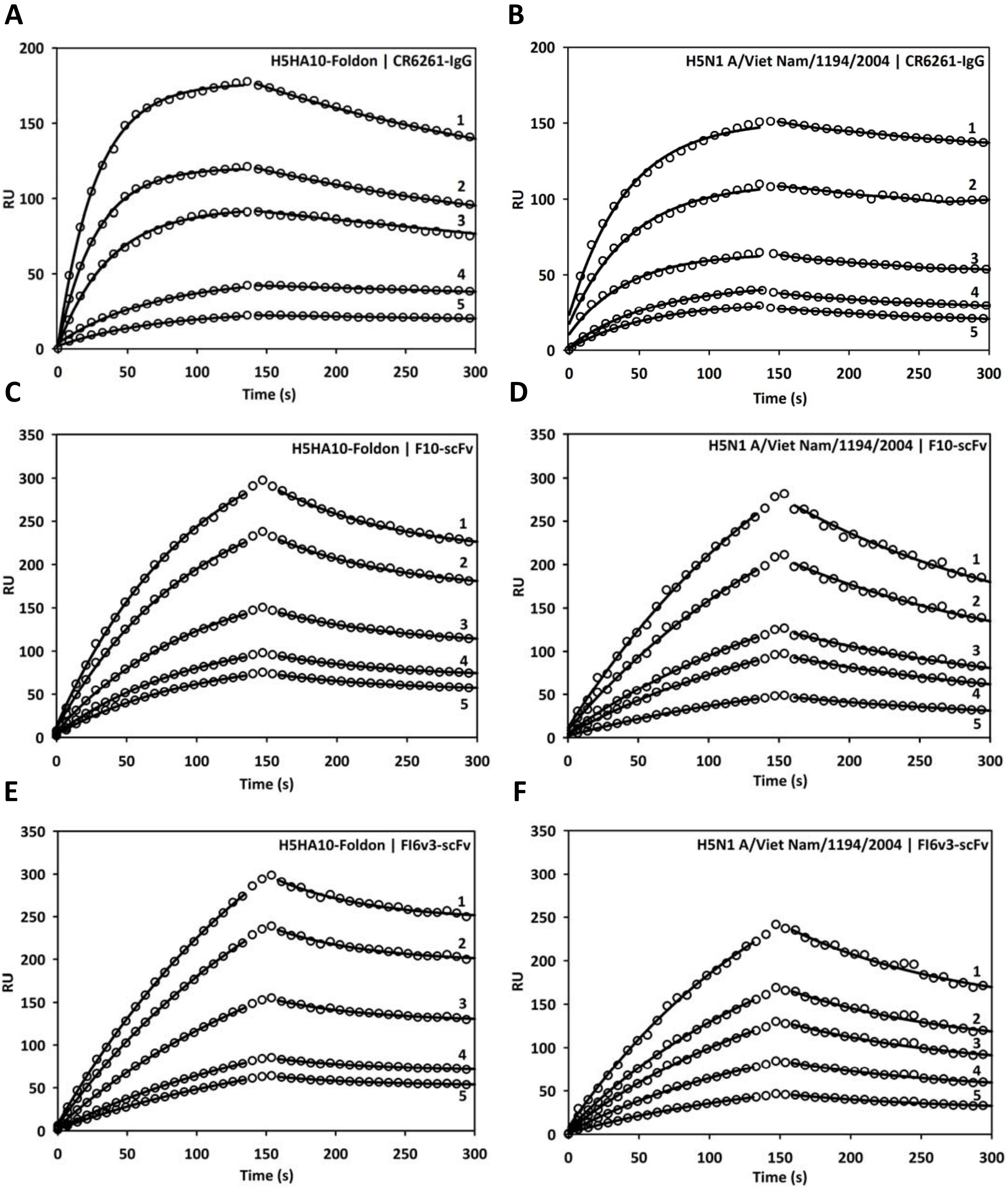

Figure S2: H5F binds HA stem-directed, conformation specific bnAbs with high affinity. SPR binding sensograms for H5F and H5 (VN/04) HA with **(A-B)** CR6261, **(C-D)** F10-scFv and **(E-F)** FI6v3-scFv. The test antibody (ligand) was immobilized (750RU) on an activated surface of a Biacore CM5 sensor chip. A concentration series of the analyte was used to obtain the kinetic parameters of binding (Supplementary Table S1). (**A, C and E)** H5F (Trace 1-5: 500nM, 250nM, 150nM, 75nM and 50nM). **(B, D and F)** H5 HA (VN/04) (Trace 1-5: 200nM, 100nM, 50nM, 25nM and 10nM). H5F bound the bnAbs with high affinity (K_D; 11.6±1.4 – 43.5±10.7nM). The kinetic parameters were obtained by fitting the data globally to a 1:1 Langmuir interaction model using BIA EVALUATION 3.1 software. The data points are in open circles, while the fits are shown as solid lines.

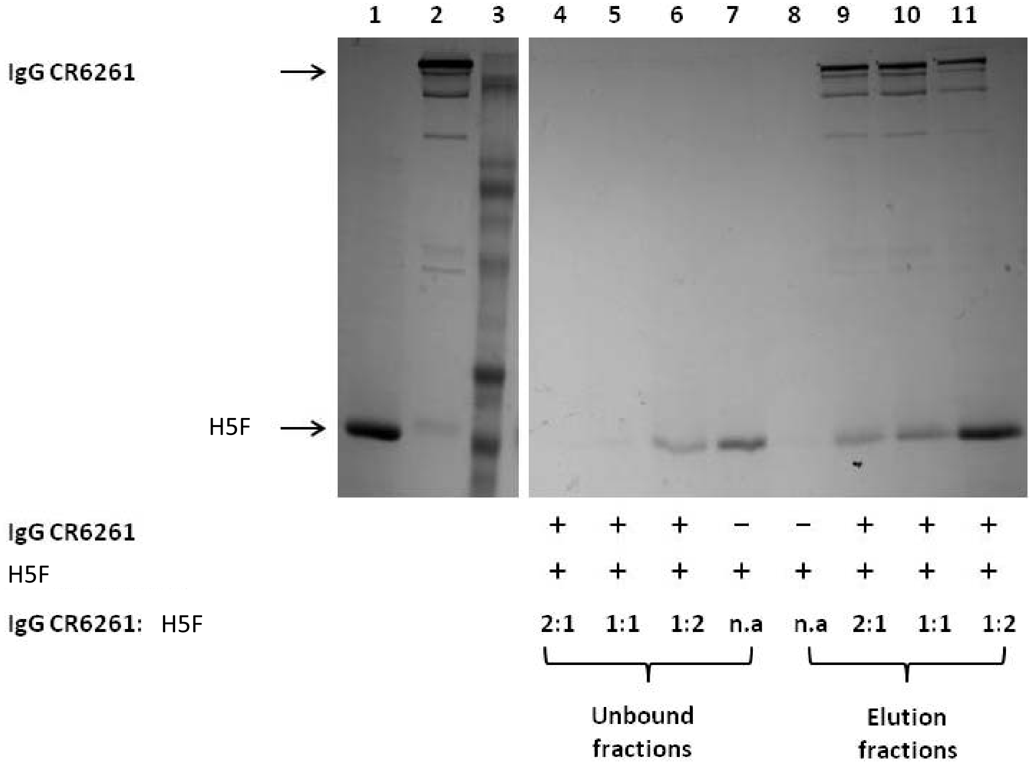

Figure S3: H5F forms a stable complex with the bnAb CR6261. Lane 1: H5F, lane 2: CR6261 (IgG), lane 3: pre-stained broad range SDS-PAGE marker (BioRad), lanes 4-7: unbound fractions, and lanes 8-11: elution fractions. H5F was incubated with CR6261 (IgG) for 2h (at 4°C) at different molar ratios (as indicated, n.a=not applicable). The protein complex (H5F with CR6261) was then pulled down using Protein G beads that bind specifically to the Fc region of a human-IgG. H5F does not bind non-specifically to Protein G beads (lane 8). The protein complex bound to the beads was eluted with 100mM glycine.HCl (pH 3) and neutralized with 1M Tris.HCl (pH 9) before SDS-PAGE analysis. All the samples were analyzed under non-reducing conditions. The gel was stained with Coomassie.

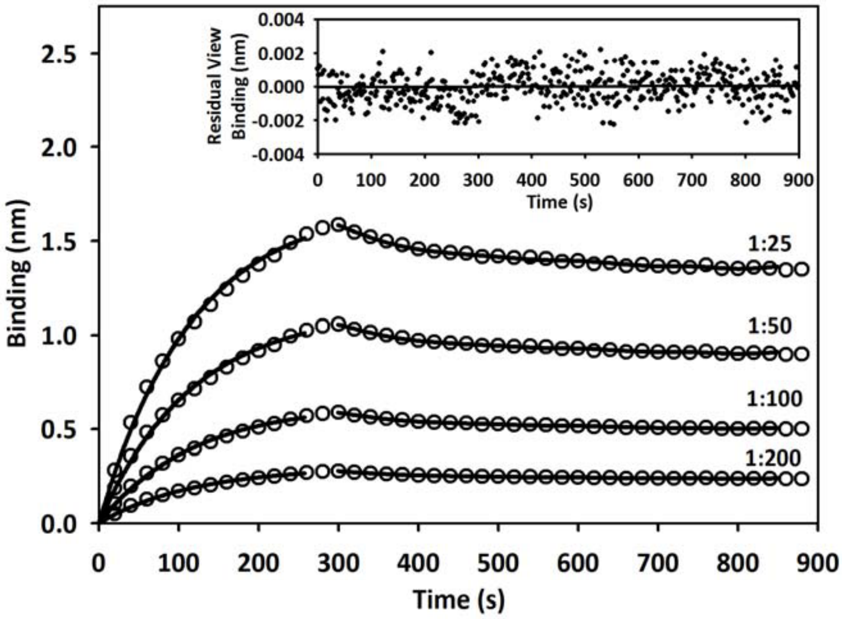

Figure S4: A representative plot demonstrating the determination of kinetic parameters for the binding of sera to HA proteins determined by BLI. The binding of sera harvested from mice vaccinated with mini-HA stem (H5F) to H5 HA (VN/04) at different sera dilutions (as indicated) has been shown. IgG was captured from the pooled mice sera using Protein G (ProG) biosensors. The biosensors loaded with the ligand were subsequently dipped in the analyte (H5 HA) wells to monitor binding. The traces were processed using the ForteBio Data Analysis Software (v8.0) and fit globally using a simple 1:1 Langmuir interaction model. The residual view (inset) indicates acceptable fits. The kinetic parameters [k_{on} ($M^{-1}s^{-1}$): 4.15×10^3, k_{off} (s^{-1}): 4.65×10^{-4}, and K_D (nM): 112.1±2.9] obtained for the binding of different serum with HA protein(s) are reported in Tables S3, S4 and S5. The kinetic parameters of binding determined by capturing the HA protein(s) on amine reactive biosensors for probing analyte interaction (serial dilutions of the sera) were comparable with the values obtained with the aforementioned experimental setup.

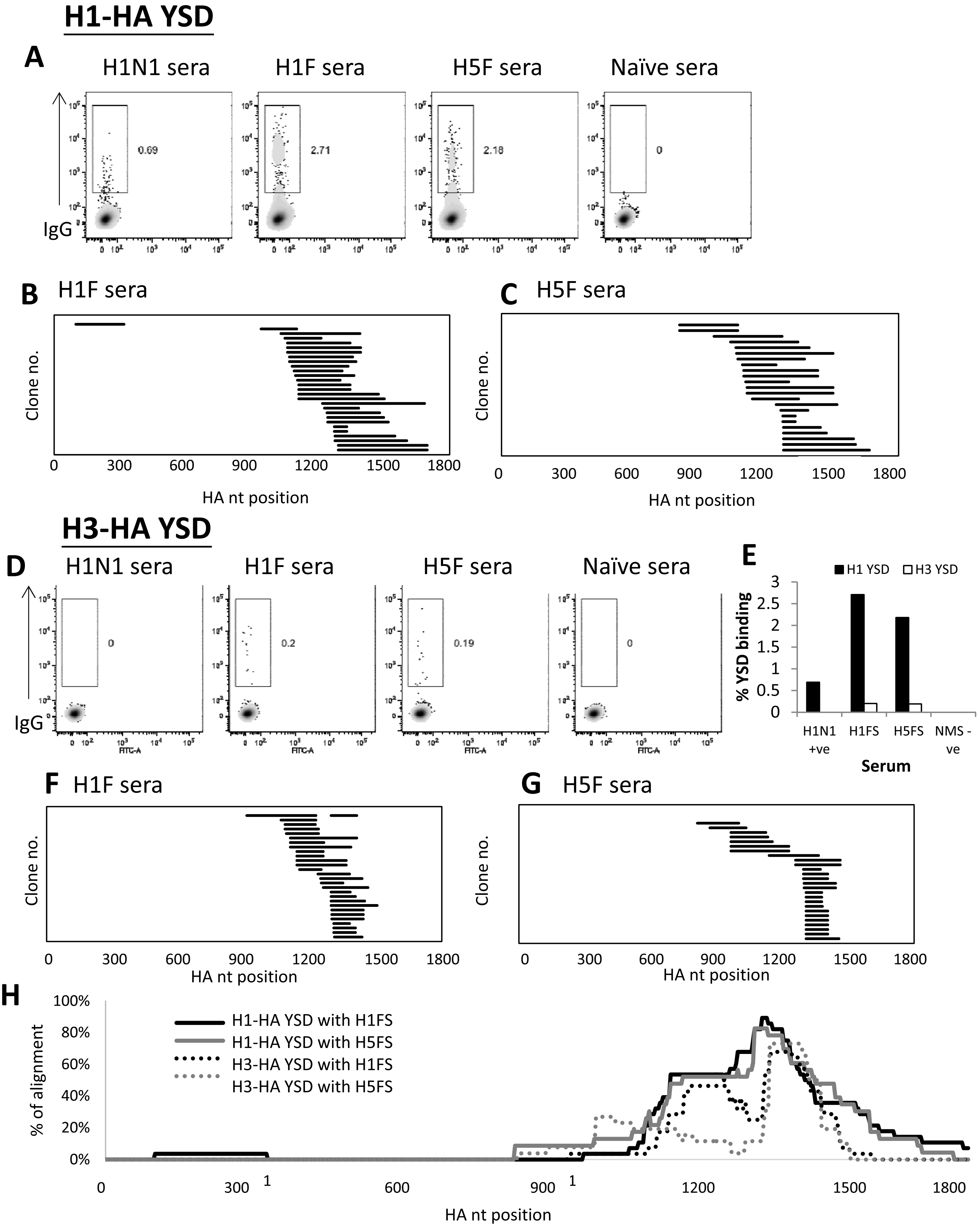

Figure S5: Mini-HA stem immunization elicits a robust antibody response against the subdominant HA2 subunit. The heat inactivated pooled sera collected from mice (n = 20) at day 21 after the secondary immunization with mini-HA stem(s) were used for screening against the YSD fragment library of unmatched influenza A **(A-C)** H1 HA (pdm Ca/09) and **(D-G)** H3 HA (HK/68). Representative FACS plots of sorted cells that were positive for HA fragment expression and sera binding (detected by anti-mouse IgG). The HA fragment of sorted yeast cells were sequenced (from 30 clones) and aligned to the consensus full length HA sequence for the H1-HA YSD for H1F sera **(B)**, H5F sera **(C)** and the H3-HA YSD for H1F sera **(F)**, H5F sera **(G)**. The % binding to the total YSD cells **(E)** (from A, D). Alignment of all HA YSD sequences **(H)** (from B, C, F, G). (number of clones sequenced H1FS H1-HA n=28, H5FS H1-HA YSD n=23, H1FS H3-HA YSD n=28, H5FS H3-HA YSD n=26).

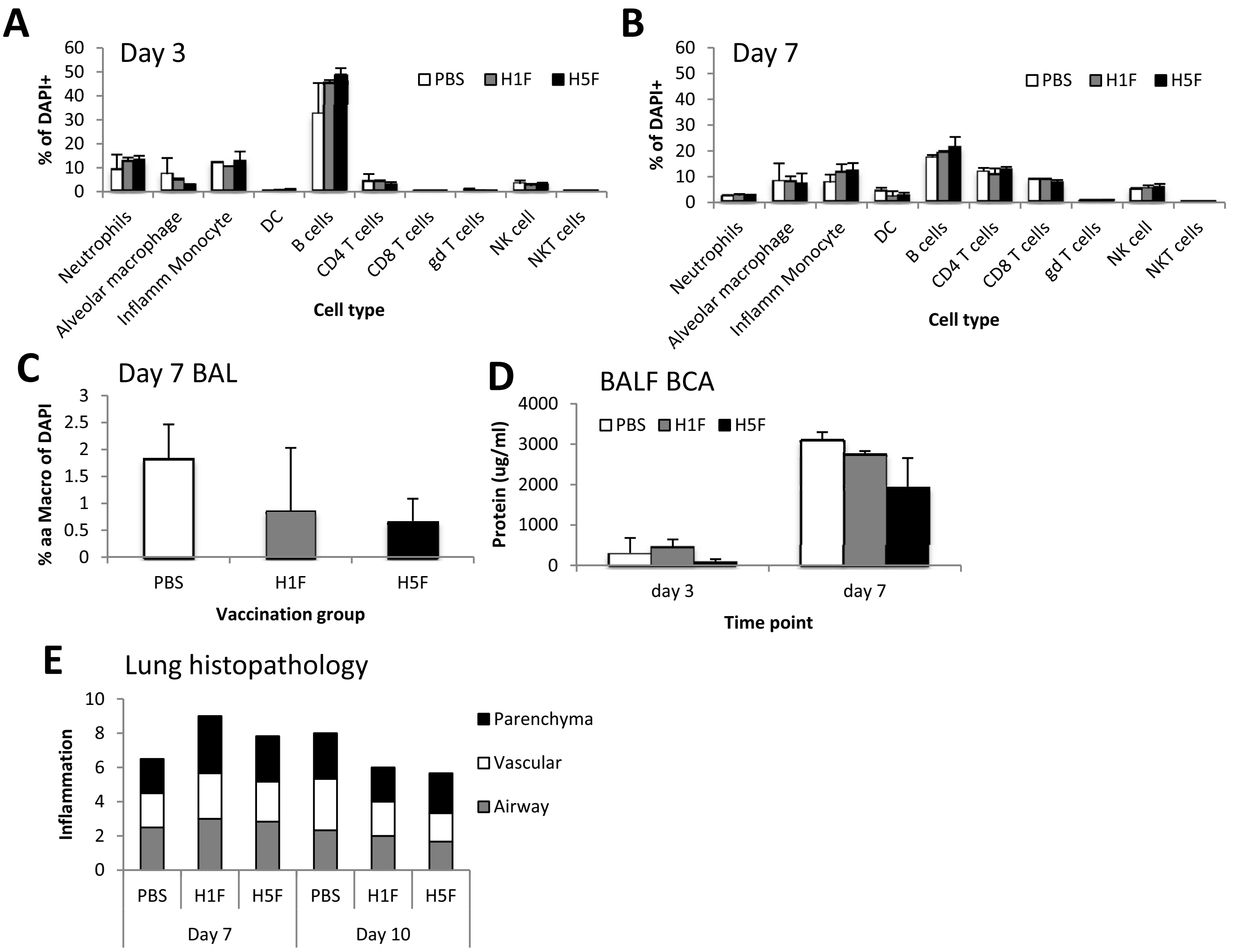

Figure S6: mini-HA stem vaccination does not alter inflammation profile after H1N1 infection despite increased survival. (A-C) The cell influx to the lung and **(D)** local lymph node of vaccinated mice after H1N1 (pdm Ca/04) infection was assessed with a panel of antibodies by flow cytometry analysis. The cell profile at (**A**) day 3 and (**B**) day 7 for innate and adaptive cell types was determined. (**C**) Alternative activated macrophages from the BAL were also determined. **(D)** The protein concentration from the BAL fluid was determined in a standard BCA protein assay at days 3 and 7 post H1N1 (pdm Ca/09) infection. **(E)** Histopathology by H&E staining of lung sections was assessed under a light microscope and vascular, airway and parenchyma inflammation scored. Data represents the mean±SD, (n=3). PBS with Addavax vaccinated mice (PBS) were used as a negative control.

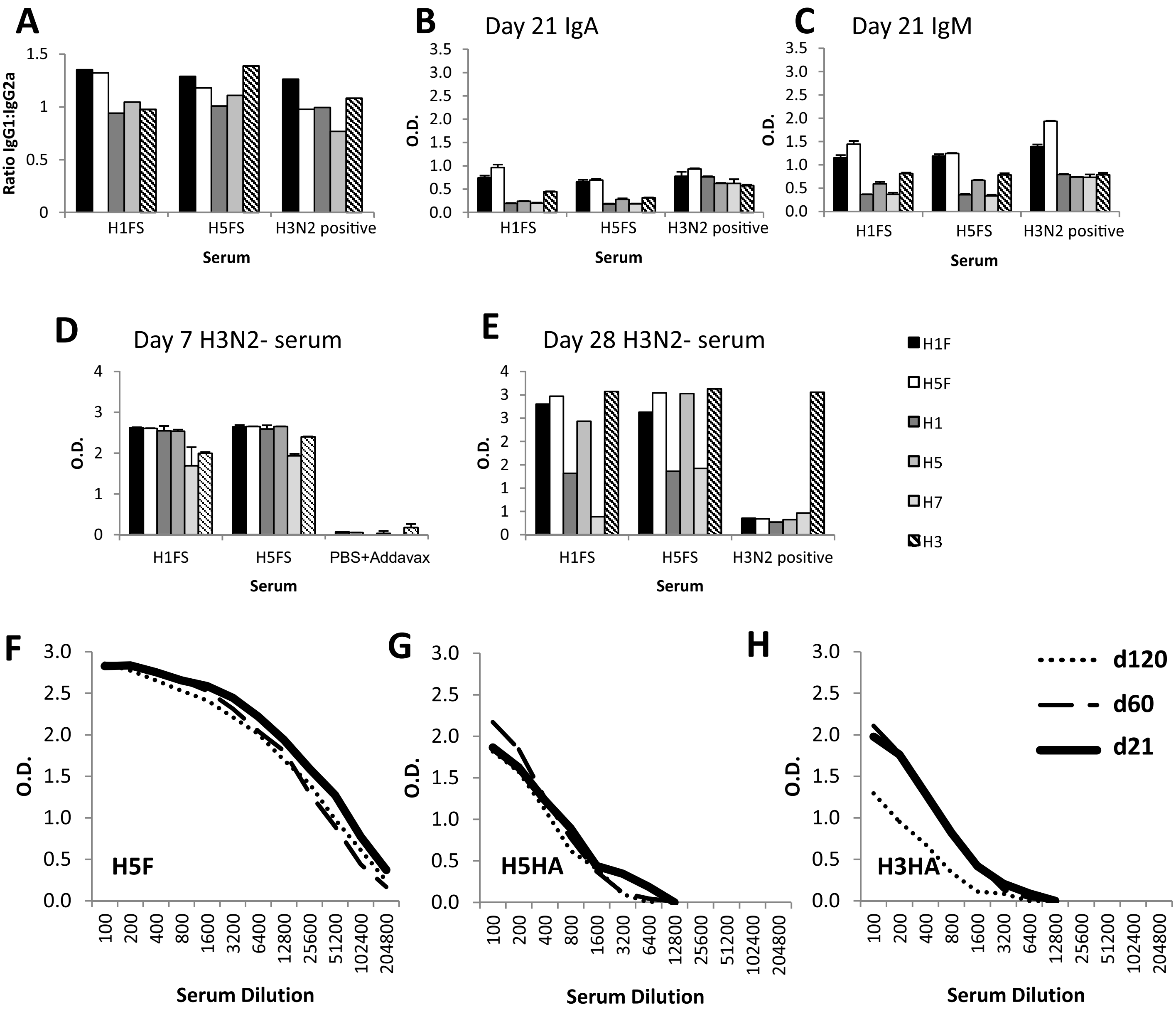

Figure S7: Mini-HA stem immunization does not skew the Th1/Th2 balance, elicits an early response after infection and is stable long term. (A) The balance between Th1/Th2 responses was evaluated by determining the ratio of IgG1/IgG2a titers to various antigens in the pooled mice sera (n=5-20) harvested at day 21 after the secondary immunization. Sera from mice recovered after a sub-lethal H3N2 (**HK/68**) virus challenge was used as a positive control. The isotype specific **(B)** IgA and **(C)** IgM cross-reactive Ab response(s) of mini-HA stem vaccinated mice sera were determined by ELISA (with serum diluted 1:100). After H3N2 infection of vaccinated mice, ELISA with recovered sera at **(D)** day 7 and **(E)** day 28 serum. **(F-H)** The longevity of mini-HA stem induced antibodies was determined in H5F sera harvested after 21, 60 and 120 days after the second vaccine dose (n=5). Antigens used in the ELISA assay were shown as indicated.
